# Reimbursement of orphan drugs: the Pompe and Fabry case in the Netherlands

**DOI:** 10.1186/1750-1172-9-S1-O17

**Published:** 2014-11-11

**Authors:** Rinke van den Brink

**Affiliations:** 1 NOS News, Hilversum, the Netherlands

## 

By the end of June 2012 NOS News got hold of a concept advice to the Minister of Health. It proposed to stop the reimbursement of the orphan drugs Myozyme against Pompe’s disease and Fabryzyme and Replagal against Fabry’s disease. All three enzyme-therapies are very expensive. The treatment for Pompe-patients costs roughly between € 400.000 and € 700.000 a year, for Fabry-patients about € 220.000 a year. The main argument for stopping the compensation was the cost-ineffectiveness of the enzyme-therapy. In other words: too much money for too little result.

The concept advice was written by the CVZ, the main advisor of the Minister on the health-insurance. The Pompe and Fabry enzyme-therapies got insurance-coverage for a limited periode of four years beginning 2009. The registration by the EMA was based on very limited evidence.

The proposal to stop the reimbursement of Pompe and Fabry therapies – only babies with the classical form of Pompe would keep the compensation - , was the first ever of this kind. There is no alternative treatment available. And no patient can afford to pay hundreds of thousands of euros year after year.

We realised that this proposal crossed a unique line. Until 2012 CVZ refused the reimbursement of several drugs. In the field of chronic diseases, for example, not every new drug is automatically reimbursed. But there are always alternatives that are. Not in this case.

We tried to publish this story with all parties concerned: the patients and their organisations, the doctors specialized in Pompe and Fabry diseases, the CVZ, the Health Ministry. They all refused to comment. For five weeks we tried to convince them, before we published without their consent.

At first the messenger was blamed for the bad news. In the end we were hailed for preventing this plan to become reality. From July 2012 until October 2013 we closely followed the decision making process. We attended hearings where everybody had the opportunity to try to convince the CVZ to change its views.

The CVZ refused to withdraw its proposal, but finally made a U-turn and advised the Minister to maintain the reimbursement of the therapies for all patients. The patients and their relatives were relieved. One could argue that common decency won.

But the real problem remains unsolved. Why are these enzyme-therapies so expensive? Why are they so mediocre? Is it true that the industry lacks incentives to come up with a better product since the limited one generates such a substantial amount of money?

